# 8-Ethyl-2-hydr­oxy-2-methyl-4-morpholinoethyl-1-thia-4-aza­spiro­[4.5]decan-3-one

**DOI:** 10.1107/S1600536808029097

**Published:** 2008-09-13

**Authors:** Mehmet Akkurt, Şerife Pınar Yalçın, Nalan Terzioğlu Klip, Orhan Büyükgüngör

**Affiliations:** a Department of Physics, Faculty of Arts and Sciences, Erciyes University, 38039 Kayseri, Turkey; b Department of Pharmacetical Chemistry, Faculty of Pharmacy, stanbul University, Beyazıt 34116, Istanbul, Turkey; c Department of Physics, Faculty of Arts and Sciences, Ondokuz Mayıs University, 55139 Samsun, Turkey

## Abstract

Mol­ecules of the title spiro­[4.5]decane derivative, C_17_H_30_N_2_O_3_S, are linked by paired O—H⋯N hydrogen bonds into centrosymmetric *R* ^2^_2_(16) dimers and these dimers are linked into a three-dimensional framework structure by C—H⋯O interactions.

## Related literature

For background on the applications of thia­zolidines, see: Babaoğlu *et al.* (2003 ▶); Pfahl *et al.* (2003 ▶); Sayyed *et al.* (2006 ▶); Sharma *et al.* (2006 ▶). For related structures, see: Akkurt *et al.* (2007 ▶); Akkurt *et al.* (2008*a* ▶,*b* ▶,*c* ▶). For ring puckering parameters, see: Cremer & Pople (1975 ▶). For hydrogen-bond motifs, see: Bernstein *et al.* (1995 ▶).
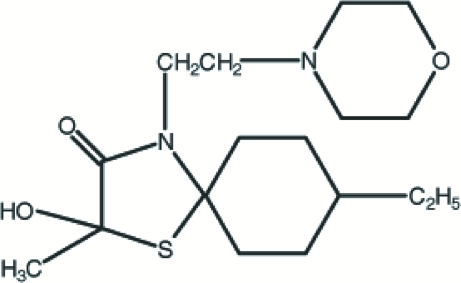


## Experimental

### 

#### Crystal data

C_17_H_30_N_2_O_3_S
*M* *_r_* = 342.50
Triclinic, 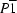

*a* = 8.1878 (4) Å
*b* = 10.2241 (5) Å
*c* = 12.2188 (6) Å
α = 79.901 (4)°
β = 73.796 (4)°
γ = 67.674 (4)°
*V* = 905.83 (8) Å^3^
*Z* = 2
Mo *K*α radiation
μ = 0.20 mm^−1^
*T* = 296 K
0.55 × 0.38 × 0.27 mm

#### Data collection

STOE IPDS II diffractometer
Absorption correction: integration (*X-RED32*; Stoe & Cie, 2002 ▶) *T*_min_ = 0.900, *T*_max_ = 0.949
18506 measured reflections
3717 independent reflections
3297 reflections with *I* > 2σ(*I*)
*R*_int_ = 0.055

#### Refinement

*R*[*F* ^2^ > 2σ(*F* ^2^)] = 0.035
*wR*(*F* ^2^) = 0.092
*S* = 1.03
3717 reflections
208 parameters
H-atom parameters constrained
Δρ_max_ = 0.25 e Å^−3^
Δρ_min_ = −0.16 e Å^−3^

### 

Data collection: *X-AREA* (Stoe & Cie, 2002 ▶); cell refinement: *X-AREA*; data reduction: *X-RED32* (Stoe & Cie, 2002 ▶); program(s) used to solve structure: *SIR97* (Altomare *et al.*, 1999 ▶); program(s) used to refine structure: *SHELXL97* (Sheldrick, 2008 ▶); molecular graphics: *ORTEP-3 for Windows* (Farrugia, 1997 ▶); software used to prepare material for publication: *WinGX* (Farrugia, 1999 ▶) and *PLATON* (Spek, 2003 ▶).

## Supplementary Material

Crystal structure: contains datablocks global, I. DOI: 10.1107/S1600536808029097/hb2794sup1.cif

Structure factors: contains datablocks I. DOI: 10.1107/S1600536808029097/hb2794Isup2.hkl

Additional supplementary materials:  crystallographic information; 3D view; checkCIF report

## Figures and Tables

**Table 1:** Hydrogen-bond geometry (Å, °)

*D*—H⋯*A*	*D*—H	H⋯*A*	*D*⋯*A*	*D*—H⋯*A*
O3—H3⋯N2^i^	0.82	1.99	2.7994 (14)	171
C9—H9*A*⋯O2^ii^	0.97	2.57	3.418 (2)	146
C15—H15*B*⋯O1^iii^	0.97	2.55	3.249 (2)	129

Symmetry codes: (i) 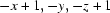; (ii) 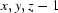; (iii) 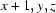.

**Fig. 1.**
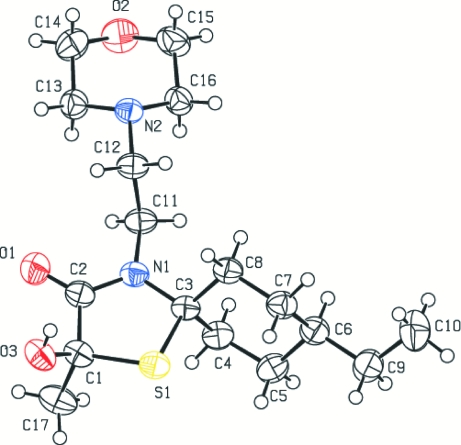
The molecular structure of (I). Displacement ellipsoids for non-H atoms are drawn at the 50% probability level.

**Fig. 2.**
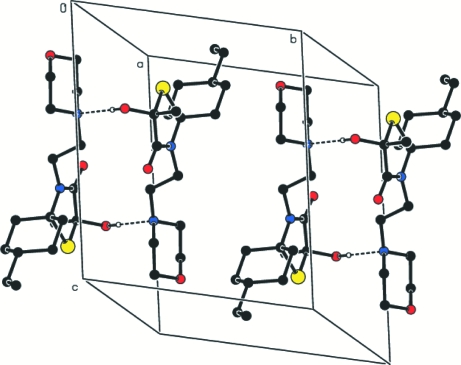
View of the hydrogen-bonded dimer in (I), forming a intermolecular R22(16) ring. For clarity, H atoms not involved in hydrogen bonds have been omitted.

## supplementary crystallographic information

### Comment

Thiazolidinone derivatives have been shown to possess antibacterial (Sayyed *et al.*, 2006), antimicrobial (Sharma *et al.*, 2006) and antimycobacterial (Babaoğlu *et al.*, 2003) activities. There have also been several reports on the anticancer properties of compounds bearing this ring system (Pfahl *et al.*, 2003).  In view of above considerations, we have synthesized the title spiro[4.5]decane derivative, (I), and we report here its crystal structure. This molecule is chiral: in the arbitrarily chosen asymmetric molecule, C1 has *R* configuration, but crystal symmetry generates a racemic mixture.

The values of the geometric parameters of the molecule shown in Fig. 1 are in their normal ranges and similar to those in the related compounds, 8-methyl-4-morpholinoethyl-1-thia-4-azaspiro[4.5]decan-3-one (Akkurt *et al.*, 2008*b*) and 2,8-dimethyl-4-morpholinoethyl-1-thia-4-azaspiro[4.5] decan-3-one (Akkurt *et al.*, 2008*c*).

In the crystal, the molecules of (I) are linked by paired O—H···N hydrogen bonds into centrosymmetric *R*^2^_2_(16) dimers and these dimers are linked into a three-dimensional framework structure by a combination of three independent C—H···O hydrogen bonds (Table 1). The thiazole ring (C1–C3/S1/N1) has an envelope conformation on S1 [puckering parameters (Cremer & Pople, 1975): Q(2) = 0.2032 (12) Å, φ(2) = 6.1 (4) °]. The cyclohexane ring (C3–C8) and morpholine ring (C13–C16/N2/O2) adopt chair conformations [puckering parameters: Q_T_ = 0.565 (2) Å, θ = 177.6 (2) °, φ = 73 (4) °, and Q_T_ = 0.570 (2) Å, θ = 0.40 (17) °, φ = 101 (16) °, respectively].

### Experimental

A mixture of morpholinoethylamin (5 mmol), 4-ethyl cyclohexanone (5 mmol) and α-mercaptopropionic acid (20 mmol) in dry benzene (20 ml) was refluxed for 18 h using a Dean–Starkwater separator. Excess solvent was evaporated *in vacuo*. The residue was taken up in chloroform. The chloroform layer was triturated with saturated NaHCO_3_ solution (2×) before drying over sodium sulfate and concentrated under reduced pressure to dryness. The crude product was triturated with diethyl ether several times and recrystallized from ethanol to yield colourless prisms of (I).

### Refinement

All H atoms were placed geometrically (C—H = 0.96–0.98 Å, O—H = 0.82 Å) and refined as riding with *U*_iso_(H) = 1.2U_eq_(C) or 1.5*U*_eq_(methyl C, O).

### Crystal data

**Table:**

C_17_H_30_N_2_O_3_S	*Z* = 2
*M**_r_* = 342.50	*F*(000) = 372
Triclinic, *P*1	*D*_x_ = 1.256 Mg m^−^^3^
Hall symbol: -P 1	Mo *K*α radiation, λ = 0.71073 Å
*a* = 8.1878 (4) Å	Cell parameters from 28660 reflections
*b* = 10.2241 (5) Å	θ = 2.2–27.4°
*c* = 12.2188 (6) Å	µ = 0.20 mm^−^^1^
α = 79.901 (4)°	*T* = 296 K
β = 73.796 (4)°	Block, colourless
γ = 67.674 (4)°	0.55 × 0.38 × 0.27 mm
*V* = 905.83 (8) Å^3^	

### Data collection

**Table:**

STOE IPDS II diffractometer	3717 independent reflections
Radiation source: sealed X-ray tube, 12 x 0.4 mm long-fine focus	3297 reflections with *I* > 2σ(*I*)
plane graphite	*R*_int_ = 0.055
Detector resolution: 6.67 pixels mm^-1^	θ_max_ = 26.5°, θ_min_ = 2.7°
ω scans	*h* = −10→10
Absorption correction: integration (X-RED32; Stoe & Cie, 2002)	*k* = −12→12
*T*_min_ = 0.900, *T*_max_ = 0.949	*l* = −15→15
18506 measured reflections	

### Refinement

**Table:**

Refinement on *F*^2^	Primary atom site location: structure-invariant direct methods
Least-squares matrix: full	Secondary atom site location: difference Fourier map
*R*[*F*^2^ > 2σ(*F*^2^)] = 0.035	Hydrogen site location: inferred from neighbouring sites
*wR*(*F*^2^) = 0.092	H-atom parameters constrained
*S* = 1.03	*w* = 1/[σ^2^(*F*_o_^2^) + (0.0459*P*)^2^ + 0.1334*P*] where *P* = (*F*_o_^2^ + 2*F*_c_^2^)/3
3717 reflections	(Δ/σ)_max_ < 0.001
208 parameters	Δρ_max_ = 0.25 e Å^−^^3^
0 restraints	Δρ_min_ = −0.16 e Å^−^^3^

### Special details

**Table:**

Geometry. Bond distances, angles *etc*. have been calculated using the rounded fractional coordinates. All su's are estimated from the variances of the (full) variance-covariance matrix. The cell e.s.d.'s are taken into account in the estimation of distances, angles and torsion angles
Refinement. Refinement on *F*^2^ for ALL reflections except those flagged by the user for potential systematic errors. Weighted *R*-factors *wR* and all goodnesses of fit *S* are based on *F*^2^, conventional *R*-factors *R* are based on *F*, with *F* set to zero for negative *F*^2^. The observed criterion of *F*^2^ > σ(*F*^2^) is used only for calculating -*R*-factor-obs *etc*. and is not relevant to the choice of reflections for refinement. *R*-factors based on *F*^2^ are statistically about twice as large as those based on *F*, and *R*-factors based on ALL data will be even larger.

### Fractional atomic coordinates and isotropic or equivalent isotropic displacement parameters (Å^2^)

**Table:**

	*x*	*y*	*z*	*U*_iso_*/*U*_eq_	
S1	0.35630 (5)	0.26913 (4)	0.21446 (3)	0.0484 (1)	
O1	0.18488 (14)	0.24461 (12)	0.54122 (9)	0.0578 (4)	
O2	0.74392 (17)	0.18404 (13)	0.83355 (9)	0.0631 (4)	
O3	0.08914 (13)	0.18609 (10)	0.34973 (9)	0.0505 (3)	
N1	0.43113 (15)	0.26810 (11)	0.40883 (9)	0.0402 (3)	
N2	0.68062 (14)	0.09805 (10)	0.64605 (9)	0.0379 (3)	
C1	0.17113 (18)	0.28560 (13)	0.34309 (12)	0.0432 (4)	
C2	0.26212 (18)	0.26248 (13)	0.44208 (11)	0.0421 (4)	
C3	0.51348 (17)	0.28899 (13)	0.28697 (10)	0.0380 (3)	
C4	0.5285 (2)	0.43629 (13)	0.25620 (12)	0.0453 (4)	
C5	0.6216 (2)	0.45605 (14)	0.13129 (12)	0.0492 (4)	
C6	0.80738 (19)	0.34126 (15)	0.09591 (12)	0.0459 (4)	
C7	0.78779 (19)	0.19575 (14)	0.12536 (12)	0.0461 (4)	
C8	0.70039 (18)	0.17493 (13)	0.25161 (11)	0.0425 (4)	
C9	0.8933 (2)	0.36576 (18)	−0.02995 (13)	0.0564 (5)	
C10	1.0855 (3)	0.2653 (2)	−0.06975 (18)	0.0795 (7)	
C11	0.52301 (19)	0.26419 (13)	0.49686 (11)	0.0433 (4)	
C12	0.61041 (19)	0.11222 (13)	0.54526 (11)	0.0422 (4)	
C13	0.53767 (18)	0.14089 (15)	0.74933 (11)	0.0463 (4)	
C14	0.6216 (2)	0.11142 (18)	0.85005 (12)	0.0582 (5)	
C15	0.8845 (2)	0.14204 (18)	0.73395 (14)	0.0568 (5)	
C16	0.80961 (18)	0.17138 (14)	0.62963 (12)	0.0440 (4)	
C17	0.0219 (2)	0.43050 (16)	0.34677 (16)	0.0596 (5)	
H3	0.16610	0.10620	0.34800	0.0760*	
H4A	0.40790	0.50770	0.27160	0.0540*	
H4B	0.59660	0.44980	0.30410	0.0540*	
H5A	0.63620	0.54780	0.11730	0.0590*	
H5B	0.54430	0.45600	0.08390	0.0590*	
H6	0.88630	0.34690	0.14080	0.0550*	
H7A	0.71400	0.18630	0.07950	0.0550*	
H7B	0.90660	0.12260	0.10690	0.0550*	
H8A	0.77890	0.17740	0.29720	0.0510*	
H8B	0.68810	0.08240	0.26710	0.0510*	
H9A	0.81850	0.35680	−0.07540	0.0680*	
H9B	0.89310	0.46220	−0.04430	0.0680*	
H10A	1.08690	0.16960	−0.05860	0.0950*	
H10B	1.16160	0.27450	−0.02650	0.0950*	
H10C	1.13010	0.28800	−0.14940	0.0950*	
H11A	0.43600	0.32010	0.55830	0.0520*	
H11B	0.61590	0.30620	0.46440	0.0520*	
H12A	0.52110	0.06600	0.56460	0.0510*	
H12B	0.70950	0.06160	0.48560	0.0510*	
H13A	0.45590	0.08870	0.76210	0.0560*	
H13B	0.46810	0.24120	0.74030	0.0560*	
H14A	0.52660	0.14080	0.91840	0.0700*	
H14B	0.68560	0.01030	0.86120	0.0700*	
H15A	0.95260	0.04150	0.74380	0.0680*	
H15B	0.96720	0.19290	0.72350	0.0680*	
H16A	0.74900	0.27260	0.61620	0.0530*	
H16B	0.90790	0.13880	0.56340	0.0530*	
H17A	−0.03000	0.44790	0.28190	0.0720*	
H17B	0.07200	0.50210	0.34490	0.0720*	
H17C	−0.07050	0.43310	0.41580	0.0720*	

### Atomic displacement parameters (Å^2^)

**Table:**

	*U*^11^	*U*^22^	*U*^33^	*U*^12^	*U*^13^	*U*^23^
S1	0.0492 (2)	0.0609 (2)	0.0430 (2)	−0.0225 (2)	−0.0195 (1)	−0.0021 (1)
O1	0.0516 (6)	0.0715 (7)	0.0478 (6)	−0.0221 (5)	−0.0079 (5)	−0.0035 (5)
O2	0.0749 (7)	0.0766 (7)	0.0507 (6)	−0.0302 (6)	−0.0222 (5)	−0.0163 (5)
O3	0.0399 (5)	0.0454 (5)	0.0692 (6)	−0.0137 (4)	−0.0166 (5)	−0.0089 (4)
N1	0.0419 (5)	0.0422 (5)	0.0398 (5)	−0.0146 (4)	−0.0161 (4)	−0.0012 (4)
N2	0.0394 (5)	0.0387 (5)	0.0373 (5)	−0.0119 (4)	−0.0133 (4)	−0.0046 (4)
C1	0.0391 (6)	0.0411 (6)	0.0511 (7)	−0.0110 (5)	−0.0166 (5)	−0.0048 (5)
C2	0.0411 (6)	0.0375 (6)	0.0459 (7)	−0.0094 (5)	−0.0131 (5)	−0.0042 (5)
C3	0.0402 (6)	0.0366 (6)	0.0392 (6)	−0.0115 (5)	−0.0158 (5)	−0.0019 (5)
C4	0.0513 (7)	0.0353 (6)	0.0488 (7)	−0.0124 (5)	−0.0145 (6)	−0.0040 (5)
C5	0.0584 (8)	0.0385 (7)	0.0504 (7)	−0.0177 (6)	−0.0146 (6)	0.0020 (5)
C6	0.0472 (7)	0.0516 (7)	0.0448 (7)	−0.0209 (6)	−0.0153 (6)	−0.0028 (5)
C7	0.0462 (7)	0.0424 (7)	0.0467 (7)	−0.0100 (6)	−0.0131 (6)	−0.0051 (5)
C8	0.0428 (7)	0.0362 (6)	0.0467 (7)	−0.0097 (5)	−0.0150 (5)	−0.0009 (5)
C9	0.0596 (9)	0.0649 (9)	0.0487 (8)	−0.0294 (8)	−0.0101 (7)	−0.0015 (7)
C10	0.0607 (10)	0.0973 (14)	0.0711 (12)	−0.0298 (10)	0.0037 (9)	−0.0090 (10)
C11	0.0521 (7)	0.0410 (6)	0.0429 (7)	−0.0155 (6)	−0.0216 (6)	−0.0030 (5)
C12	0.0495 (7)	0.0392 (6)	0.0417 (6)	−0.0123 (5)	−0.0192 (6)	−0.0059 (5)
C13	0.0437 (7)	0.0516 (7)	0.0431 (7)	−0.0171 (6)	−0.0075 (6)	−0.0058 (5)
C14	0.0662 (9)	0.0691 (10)	0.0389 (7)	−0.0239 (8)	−0.0104 (7)	−0.0058 (6)
C15	0.0512 (8)	0.0691 (10)	0.0597 (9)	−0.0224 (7)	−0.0249 (7)	−0.0069 (7)
C16	0.0411 (6)	0.0489 (7)	0.0451 (7)	−0.0175 (6)	−0.0114 (5)	−0.0050 (5)
C17	0.0541 (8)	0.0448 (8)	0.0783 (11)	−0.0033 (6)	−0.0299 (8)	−0.0094 (7)

### Geometric parameters (Å, °)

**Table:**

S1—C1	1.8347 (15)	C5—H5A	0.9700
S1—C3	1.8386 (15)	C5—H5B	0.9700
O1—C2	1.2179 (17)	C6—H6	0.9800
O2—C14	1.412 (2)	C7—H7A	0.9700
O2—C15	1.420 (2)	C7—H7B	0.9700
O3—C1	1.3971 (18)	C8—H8A	0.9700
O3—H3	0.8200	C8—H8B	0.9700
N1—C2	1.350 (2)	C9—H9A	0.9700
N1—C11	1.4623 (19)	C9—H9B	0.9700
N1—C3	1.4680 (16)	C10—H10A	0.9600
N2—C12	1.4620 (18)	C10—H10B	0.9600
N2—C16	1.466 (2)	C10—H10C	0.9600
N2—C13	1.4609 (18)	C11—H11A	0.9700
C1—C17	1.519 (2)	C11—H11B	0.9700
C1—C2	1.531 (2)	C12—H12A	0.9700
C3—C4	1.5300 (18)	C12—H12B	0.9700
C3—C8	1.5299 (19)	C13—H13A	0.9700
C4—C5	1.520 (2)	C13—H13B	0.9700
C5—C6	1.527 (2)	C14—H14A	0.9700
C6—C9	1.522 (2)	C14—H14B	0.9700
C6—C7	1.526 (2)	C15—H15A	0.9700
C7—C8	1.5237 (19)	C15—H15B	0.9700
C9—C10	1.513 (3)	C16—H16A	0.9700
C11—C12	1.5314 (18)	C16—H16B	0.9700
C13—C14	1.504 (2)	C17—H17A	0.9600
C15—C16	1.501 (2)	C17—H17B	0.9600
C4—H4A	0.9700	C17—H17C	0.9600
C4—H4B	0.9700		
			
C1—S1—C3	94.89 (6)	C8—C7—H7B	109.00
C14—O2—C15	110.20 (12)	H7A—C7—H7B	108.00
C1—O3—H3	109.00	C3—C8—H8A	109.00
C2—N1—C3	120.17 (12)	C3—C8—H8B	109.00
C3—N1—C11	121.19 (12)	C7—C8—H8A	109.00
C2—N1—C11	118.47 (11)	C7—C8—H8B	109.00
C12—N2—C13	113.18 (12)	H8A—C8—H8B	108.00
C13—N2—C16	109.95 (11)	C6—C9—H9A	109.00
C12—N2—C16	113.51 (11)	C6—C9—H9B	109.00
S1—C1—C2	104.46 (10)	C10—C9—H9A	109.00
S1—C1—C17	113.16 (10)	C10—C9—H9B	109.00
O3—C1—C2	112.49 (11)	H9A—C9—H9B	108.00
O3—C1—C17	106.76 (13)	C9—C10—H10A	109.00
C2—C1—C17	109.29 (12)	C9—C10—H10B	109.00
S1—C1—O3	110.80 (9)	C9—C10—H10C	109.00
O1—C2—N1	124.16 (14)	H10A—C10—H10B	109.00
N1—C2—C1	113.68 (11)	H10A—C10—H10C	109.00
O1—C2—C1	122.14 (14)	H10B—C10—H10C	109.00
S1—C3—N1	103.71 (10)	N1—C11—H11A	109.00
S1—C3—C4	110.66 (10)	N1—C11—H11B	109.00
N1—C3—C4	111.72 (10)	C12—C11—H11A	109.00
N1—C3—C8	111.49 (10)	C12—C11—H11B	109.00
S1—C3—C8	109.19 (9)	H11A—C11—H11B	108.00
C4—C3—C8	109.91 (12)	N2—C12—H12A	108.00
C3—C4—C5	111.93 (11)	N2—C12—H12B	108.00
C4—C5—C6	113.29 (12)	C11—C12—H12A	108.00
C5—C6—C7	109.26 (13)	C11—C12—H12B	108.00
C5—C6—C9	110.96 (12)	H12A—C12—H12B	107.00
C7—C6—C9	112.66 (12)	N2—C13—H13A	110.00
C6—C7—C8	111.36 (11)	N2—C13—H13B	110.00
C3—C8—C7	112.03 (11)	C14—C13—H13A	110.00
C6—C9—C10	114.58 (14)	C14—C13—H13B	110.00
N1—C11—C12	111.58 (11)	H13A—C13—H13B	108.00
N2—C12—C11	115.86 (11)	O2—C14—H14A	109.00
N2—C13—C14	109.49 (13)	O2—C14—H14B	109.00
O2—C14—C13	111.30 (12)	C13—C14—H14A	109.00
O2—C15—C16	111.43 (14)	C13—C14—H14B	109.00
N2—C16—C15	109.68 (12)	H14A—C14—H14B	108.00
C3—C4—H4A	109.00	O2—C15—H15A	109.00
C3—C4—H4B	109.00	O2—C15—H15B	109.00
C5—C4—H4A	109.00	C16—C15—H15A	109.00
C5—C4—H4B	109.00	C16—C15—H15B	109.00
H4A—C4—H4B	108.00	H15A—C15—H15B	108.00
C4—C5—H5A	109.00	N2—C16—H16A	110.00
C4—C5—H5B	109.00	N2—C16—H16B	110.00
C6—C5—H5A	109.00	C15—C16—H16A	110.00
C6—C5—H5B	109.00	C15—C16—H16B	110.00
H5A—C5—H5B	108.00	H16A—C16—H16B	108.00
C5—C6—H6	108.00	C1—C17—H17A	109.00
C7—C6—H6	108.00	C1—C17—H17B	109.00
C9—C6—H6	108.00	C1—C17—H17C	109.00
C6—C7—H7A	109.00	H17A—C17—H17B	109.00
C6—C7—H7B	109.00	H17A—C17—H17C	110.00
C8—C7—H7A	109.00	H17B—C17—H17C	109.00
			
C3—S1—C1—O3	−137.43 (10)	C12—N2—C16—C15	−175.82 (11)
C3—S1—C1—C2	−16.07 (9)	C17—C1—C2—O1	70.12 (17)
C3—S1—C1—C17	102.70 (12)	C17—C1—C2—N1	−108.05 (14)
C1—S1—C3—N1	14.83 (9)	S1—C1—C2—N1	13.31 (13)
C1—S1—C3—C4	−105.10 (10)	S1—C1—C2—O1	−168.51 (11)
C1—S1—C3—C8	133.79 (9)	O3—C1—C2—N1	133.56 (12)
C14—O2—C15—C16	58.82 (17)	O3—C1—C2—O1	−48.27 (17)
C15—O2—C14—C13	−59.22 (17)	C4—C3—C8—C7	−55.31 (15)
C11—N1—C3—C4	−65.77 (16)	N1—C3—C8—C7	−179.75 (12)
C2—N1—C11—C12	81.87 (15)	S1—C3—C4—C5	−67.62 (15)
C11—N1—C2—O1	−5.22 (19)	N1—C3—C4—C5	177.37 (13)
C3—N1—C2—C1	−2.34 (16)	S1—C3—C8—C7	66.25 (13)
C2—N1—C3—C4	109.35 (14)	C8—C3—C4—C5	53.06 (16)
C3—N1—C11—C12	−102.93 (14)	C3—C4—C5—C6	−54.41 (18)
C11—N1—C3—C8	57.65 (15)	C4—C5—C6—C7	54.72 (17)
C11—N1—C2—C1	172.91 (10)	C4—C5—C6—C9	179.53 (13)
C3—N1—C2—O1	179.53 (12)	C5—C6—C7—C8	−55.82 (16)
C11—N1—C3—S1	175.01 (9)	C5—C6—C9—C10	174.32 (15)
C2—N1—C3—C8	−127.23 (13)	C7—C6—C9—C10	−62.8 (2)
C2—N1—C3—S1	−9.86 (13)	C9—C6—C7—C8	−179.64 (13)
C13—N2—C16—C15	56.27 (14)	C6—C7—C8—C3	57.90 (17)
C12—N2—C13—C14	175.30 (11)	N1—C11—C12—N2	−170.62 (12)
C16—N2—C13—C14	−56.60 (14)	N2—C13—C14—O2	58.47 (16)
C13—N2—C12—C11	71.56 (15)	O2—C15—C16—N2	−57.46 (16)
C16—N2—C12—C11	−54.67 (16)		

### Hydrogen-bond geometry (Å, °)

**Table:**

*D*—H···*A*	*D*—H	H···*A*	*D*···*A*	*D*—H···*A*
O3—H3···N2^i^	0.82	1.99	2.7994 (14)	171
C9—H9A···O2^ii^	0.97	2.57	3.418 (2)	146
C15—H15B···O1^iii^	0.97	2.55	3.249 (2)	129

Symmetry codes: (i) −*x*+1, −*y*, −*z*+1; (ii) *x*, *y*, *z*−1; (iii) *x*+1, *y*, *z*.
